# Two leucine-rich repeat receptor-like kinases initiate herbivory defense responses in tea plants

**DOI:** 10.1093/hr/uhae281

**Published:** 2024-10-02

**Authors:** Qi Jiang, Changqing Ding, Lingjia Feng, Zhenwei Wu, Yujie Liu, Lintong He, Chuande Liu, Lu Wang, Jianming Zeng, Jianyan Huang, Meng Ye

**Affiliations:** Key Laboratory of Biology, Genetics and Breeding of Special Economic Animals and Plants, Ministry of Agriculture and Rural Affairs, National Center for Tea Plant Improvement, Tea Research Institute, Chinese Academy of Agricultural Sciences, 9 South Meiling Road, Hangzhou 310008, China; Key Laboratory of Biology, Genetics and Breeding of Special Economic Animals and Plants, Ministry of Agriculture and Rural Affairs, National Center for Tea Plant Improvement, Tea Research Institute, Chinese Academy of Agricultural Sciences, 9 South Meiling Road, Hangzhou 310008, China; Key Laboratory of Biology, Genetics and Breeding of Special Economic Animals and Plants, Ministry of Agriculture and Rural Affairs, National Center for Tea Plant Improvement, Tea Research Institute, Chinese Academy of Agricultural Sciences, 9 South Meiling Road, Hangzhou 310008, China; Key Laboratory of Biology, Genetics and Breeding of Special Economic Animals and Plants, Ministry of Agriculture and Rural Affairs, National Center for Tea Plant Improvement, Tea Research Institute, Chinese Academy of Agricultural Sciences, 9 South Meiling Road, Hangzhou 310008, China; Key Laboratory of Biology, Genetics and Breeding of Special Economic Animals and Plants, Ministry of Agriculture and Rural Affairs, National Center for Tea Plant Improvement, Tea Research Institute, Chinese Academy of Agricultural Sciences, 9 South Meiling Road, Hangzhou 310008, China; Key Laboratory of Biology, Genetics and Breeding of Special Economic Animals and Plants, Ministry of Agriculture and Rural Affairs, National Center for Tea Plant Improvement, Tea Research Institute, Chinese Academy of Agricultural Sciences, 9 South Meiling Road, Hangzhou 310008, China; Key Laboratory of Biology, Genetics and Breeding of Special Economic Animals and Plants, Ministry of Agriculture and Rural Affairs, National Center for Tea Plant Improvement, Tea Research Institute, Chinese Academy of Agricultural Sciences, 9 South Meiling Road, Hangzhou 310008, China; Key Laboratory of Biology, Genetics and Breeding of Special Economic Animals and Plants, Ministry of Agriculture and Rural Affairs, National Center for Tea Plant Improvement, Tea Research Institute, Chinese Academy of Agricultural Sciences, 9 South Meiling Road, Hangzhou 310008, China; Key Laboratory of Biology, Genetics and Breeding of Special Economic Animals and Plants, Ministry of Agriculture and Rural Affairs, National Center for Tea Plant Improvement, Tea Research Institute, Chinese Academy of Agricultural Sciences, 9 South Meiling Road, Hangzhou 310008, China; Key Laboratory of Biology, Genetics and Breeding of Special Economic Animals and Plants, Ministry of Agriculture and Rural Affairs, National Center for Tea Plant Improvement, Tea Research Institute, Chinese Academy of Agricultural Sciences, 9 South Meiling Road, Hangzhou 310008, China; Key Laboratory of Biology, Genetics and Breeding of Special Economic Animals and Plants, Ministry of Agriculture and Rural Affairs, National Center for Tea Plant Improvement, Tea Research Institute, Chinese Academy of Agricultural Sciences, 9 South Meiling Road, Hangzhou 310008, China

## Abstract

Leucine-rich repeat receptor-like kinases (LRR-RLKs) have emerged as key regulators of herbivory perception and subsequent defense initiation. While their functions in grass plants have been gradually elucidated, the roles of herbivory-related LRR-RLKs in woody plants remain largely unknown. In this study, we mined the genomic and transcriptomic data of tea plants (*Camellia sinensis*) and identified a total of 307 CsLRR-RLK members. Phylogenetic analysis grouped these CsLRR-RLKs into 14 subgroups along with their Arabidopsis homologs. Gene structure and conserved domain analyses revealed notable similarities among subgroup members. Among the identified CsLRR-RLKs, we focused on two plasma membrane-localized LRR-RLKs, CsLRR-RLK44, and CsLRR-RLK239, which do not form homodimers or heterodimers with each other. Both respond strongly to herbivory, and their expression patterns significantly correlate with herbivore resistance phenotypes across different tea accessions. CsLRR-RLK44 and CsLRR-RLK239 act upstream of mitogen-activated protein kinase (MPK) cascades and modulate the expression of defense-related MPKs and WRKY transcription factors. Additionally, silencing *CsLRR-RLK44* or *CsLRR-RLK239* reduced the levels of herbivory-induced jasmonates, thereby weakening the plant resistance to tea geometrid larvae (*Ectropis obliqua*). Our work is the first to demonstrate that in woody plants, LRR-RLKs are essential for enhancing herbivore resistance through the activation of the canonical signaling, including MPKs, WRKYs, and jasmonates. Furthermore, our study extends mechanistic insights into how LRR-RLKs initiate plant defenses from grasses to economically important tree species.

## Introduction

Plants encounter numerous abiotic and biotic stresses in nature, with insect herbivores posing one of the most significant threats to their survival [1, 2]. Upon herbivore attack, plants dramatically activate their defense signaling to produce effective resistance. The initiation of this defense response starts with the perception of herbivory, which is achieved through the detection of elicitor compounds derived from herbivory, such as damage-associated molecular patterns (DAMPs) and herbivore-associated molecular patterns (HAMPs) [3]. This perception process likely involves a group of membrane-bound receptors analogous to those involved in recognizing microbe-associated molecular patterns (MAMP) [4].

To date, the molecular basis of herbivory perception and subsequent defense activation is still not fully understood. However, accumulating evidence points to the involvement of leucine-rich repeat receptor-like kinases (LRR-RLKs) in plant responses to herbivory [3]. Comprehensive genome-wide analyses of LRR-RLKs have been conducted in various species, including Arabidopsis (*Arabidopsis thaliana*) [5], rice (*Oryza sativa*) [6], maize (*Zea mays*) [7], wheat (*Triticum aestivum*) [8], tomato (*Solanum lycopersicum*) [9], common bean (*Phaseolus vulgaris*) [10], peanut (*Arachis hypogaea*) [11], poplar (*Populus trichocarpa*) [12], and masson pine (*Pinus massoniana*) [13], among others. A typical LRR-RLK protein comprises an extracellular domain with repeated LRR motifs, a transmembrane domain, and an intracellular kinase domain [14]. One notable study showed that rice OsLRR-RLK1 serves as a candidate receptor for HAMPs, initiating early defense signaling against rice-chewing herbivores [15]. In maize, the homologous gene *ZmFAC* has been implicated in sensing fatty acid–amino acid conjugates present in the oral secretions of the armyworm *Spodoptera exigua*, thereby enhancing defense responses [16]. Similarly, soybean GmHAK1 responds to polysaccharide signals from the herbivore *Spodoptera litura*, playing a crucial role in triggering host plant defenses [17]. Apart from LRR-RLKs, receptor-like proteins (RLPs), which lack the protein kinase domain of LRR-RLKs, are also involved in herbivory detection. For instance, the inceptin receptor INR in cowpea is responsible for detecting elicitors and enhancing defense against *S. exigua* [18].

While only a limited number of LRR-RLKs have been identified as potential players in herbivory perception and defense initiation, the downstream defense signaling coordinated by LRR-RLKs is becoming evident [3, 19]. The emerging view is that after perceiving HAMPs or DAMPs, LRR-RLKs activate the early defense signaling cascades, including mitogen-activated protein kinases (MPKs), calcium influx, and so forth [19]. These active cascades then activate transcription factors, including WRKYs. For instance, in rice, OsLRR-RLK1 and OsLRR-RLK2 positively regulate the expression and activity of MPKs like OsMPK3 and OsMPK6, which subsequently modulate the expression of WRKY transcription factors that interact with MPKs [15, 20]. These activated transcription factors bind to specific *cis*-elements in the promoters of target genes, thereby influencing hormonal signaling networks including jasmonic acid (JA), salicylic acid (SA), and reactive oxygen species (ROS) [21]. These defense signaling pathways result in specific defense responses to different types of herbivores. For instance, in Arabidopsis (*A. thaliana*), knockout of the LRR-RLK *BAK1* reduces the induction of ROS in response to green peach aphid elicitors, compromising plant resistance to aphid attack [22]. While silencing *BAK1* in tobacco (*Nicotiana attenuata*) affects the burst of JA and JA-Ile elicited by mechanical damage or oral secretions, it does not influence herbivore performance [23]. The Arabidopsis *pepr1pepr2* double mutant shows decreased accumulation of JA in response to oral secretions and reduced resistance to *Spodoptera littoralis* [24]. Despite the promising progress of LRR-RLKs in regulation of defense responses to herbivory, to the best of our knowledge, the herbivory-related LRR-RLKs in woody plants are still unknown, let alone their molecular mechanisms in initiating defense responses against herbivores.

Tea plant (*Camellia sinensis*), an important economic crop in China, suffers heavily from insect herbivores [25, 26]. Preliminary transcriptomic analysis indicates that feeding by tea geometrid caterpillars alters the expression of 10 LRR-RLKs [27], suggesting their potential involvement in tea plant defense mechanisms against herbivores. Thus, tea plants serve as an ideal model to unravel how LRR-RLKs function in plant–herbivore interactions within woody species. In this study, we mined the genome and transcriptome data to catalog all LRR-RLK genes of tea plants. By assessing the correlation between LRR-RLK expression patterns and resistance phenotypes across different tea accessions, we identified two key LRR-RLKs, *CsLRR-RLK44* and *CsLRR-RLK239*. Employing reverse genetic approach, we further elucidated how these two LRR-RLKs initiate defenses against a destructive insect pest, tea geometrid (*Ectropis obliqua*). Our study underscores the pivotal role of LRR-RLKs in orchestrating defense signaling and enhancing resistance against herbivores in an economically important tree species. These findings significantly contribute to our understanding of the evolutionary conservation of early defense signaling initiation in response to herbivory.

## Results

### Genome-wide identification of *CsLRR-RLKs*

**Figure 1:**
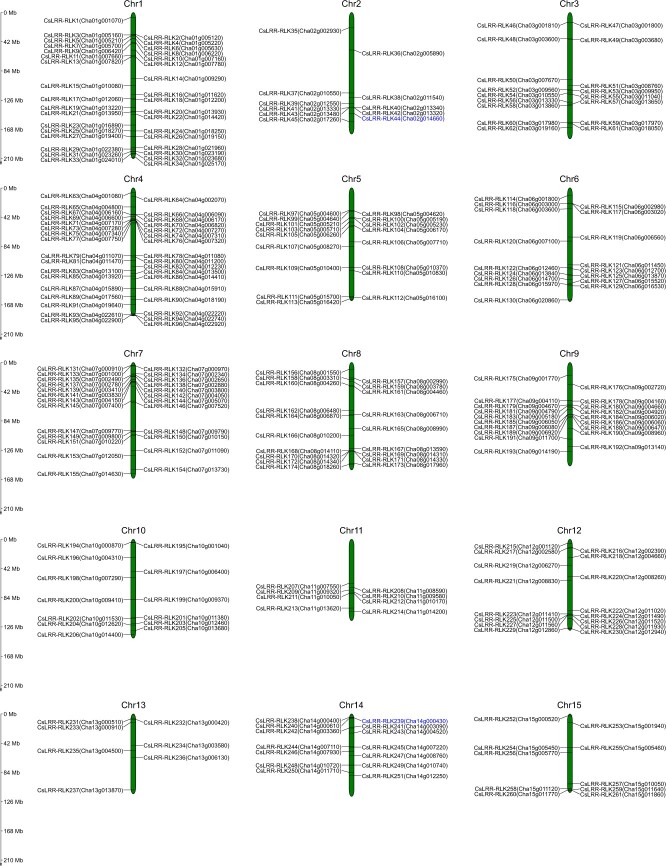
**Chromosomal distribution of *CsLRR-RLK* genes in tea plants.** A total of 261 *CsLRR-RLKs* were mapped onto 15 chromosomes, with each chromosome indicated at the top. The positions of *CsLRR-RLKs* on the chromosomes are denoted by horizontal lines. The values on the scale of the vertical lines on the left represent the physical distance in megabases (Mb). The two LRR-RLKs CsLRR-RLK44 and CsLRR-RLK239 were selected for further analysis to assess their regulatory roles in plant defense signaling and herbivore resistance.

To achieve a comprehensive understanding of LRR-RLKs in tea plants, we systematically mined the genome of the Longjing 43 (LJ43) cultivar for LRR-RLK candidate genes. We identified a total of 307 *CsLRR-RLK* genes, each confirmed to contain at least one LRR motif, a transmembrane region, and a kinase domain. These genes were named and numbered based on their respective chromosome locations, from *CsLRR-RLK1* to *CsLRR-RLK307* (Table S1). Chromosomal mapping revealed that 261 out of the 307 *CsLRR-RLK* genes were distributed across all 15 chromosomes (chromosomes 1–15), while the remaining 46 members were located on unassembled genomic sequence scaffolds (ChrUn) (Fig. 1, Table S1). Notably, the distribution of *CsLRR-RLK* members was uneven across chromosomes, with Chr1 and Chr4 harboring the most (34 genes each), and Chr13 containing the fewest (7 genes) (Fig. 1, Table S1).

The protein sequences encoded by these *CsLRR-RLK* genes exhibited significant diversity. They varied in length from 341 to 2126 amino acids, corresponding to molecular weights spanning from 37.58 to 233.17 kDa. On average, these proteins consisted of ~877 amino acids. Additionally, the theoretical isoelectric points of CsLRR-RLK proteins exhibited notable variability, spanning from 4.98 to 9.59 (Table S1).

### Phylogenetic and structure analysis of *CsLRR-RLKs*

**Figure 2:**
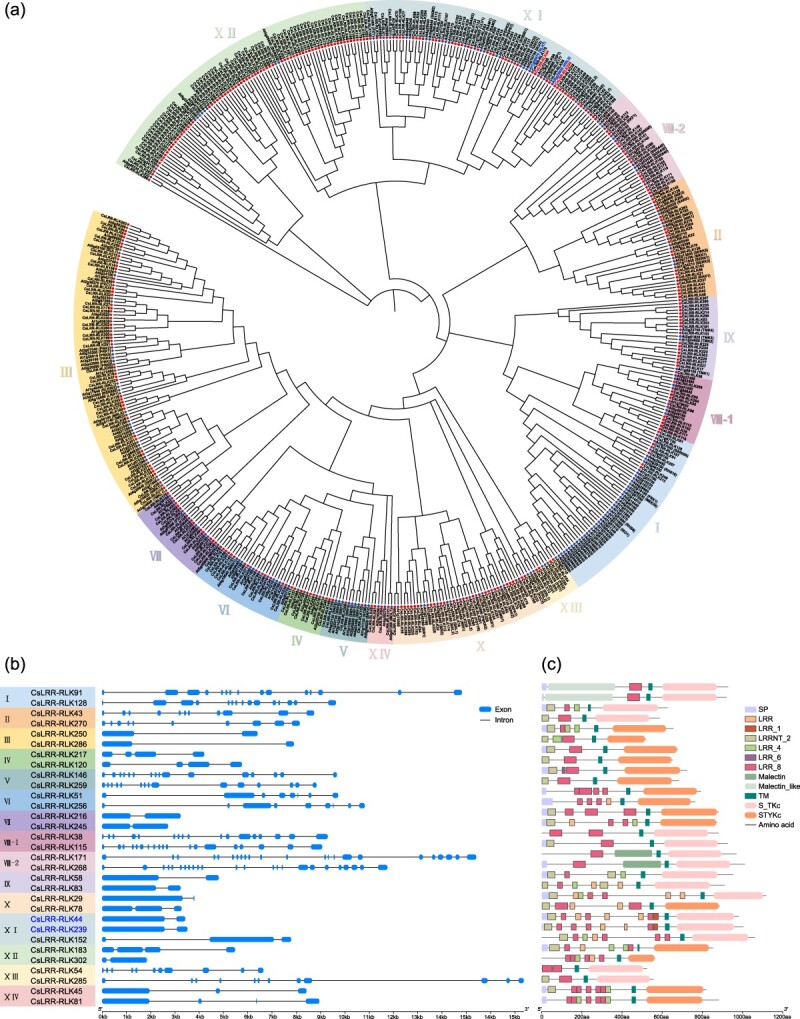
**Phylogenetic and structure analysis of *CsLRR-RLKs*. (a)** Phylogenetic tree depicting the relationships among LRR-RLKs from tea and Arabidopsis plants. This analysis includes 307 CsLRR-RLKs and 223 AtLRR-RLKs, divided into 14 distinct subgroups, each visually distinguished by a unique background color for clarity. The tree was constructed in MEGA 7.0 using the Neighbor-Joining method with 1000 bootstrap replicates. **(b)** Gene structure of *CsLRR-RLKs* in tea plants, showing intron (lines) and exon (filled boxes) patterns. The horizontal line below represents the gene length. The two LRR-RLKs CsLRR-RLK44 and CsLRR-RLK239 were selected for further analysis to assess their regulatory roles in plant defense signaling and herbivore resistance. **(c)** Conserved domains in CsLRR-RLK proteins, represented by different colored boxes: signal peptide (SP), various types of LRRs, transmembrane domain (TM), and distinct types of kinase domains (S_TKc: Ser/Thr kinase; STYKc: Ser/Thr/Tyr kinase). The horizontal line below indicates the protein length.

To elucidate the evolutionary connections among the 307 CsLRR-RLKs, we constructed a phylogenetic tree incorporating LRR-RLKs from both tea and Arabidopsis. The resulting tree distinctly clustered all CsLRR-RLKs into 14 major groups, aligned with the nomenclature of Arabidopsis homologs within the same groups (I–XIV) (Fig. 2a, Table S2) [5]. Notably, Subgroup XII contained the highest membership, with 73 proteins, while Subgroup V, XIII, and XIV contained the fewest, with only 4 proteins, respectively (Fig. 2a, Table S2). Although most subgroups contained members from both tea and Arabidopsis, substantial differences in gene counts were observed. For example, Subgroup XII in tea plants comprised ~24% of all CsLRR-RLKs, ~5.3 times the number found in Arabidopsis. Conversely, Subgroup I, the largest in Arabidopsis, contained over 8 times the number of genes compared to tea plants (Table S3). These findings confirm the subclassification of the 307 CsLRR-RLKs and suggest functional similarities between CsLRR-RLKs and their Arabidopsis orthologs, shedding light on the evolution and diversity of gene families in tea plants.

**Figure 3:**
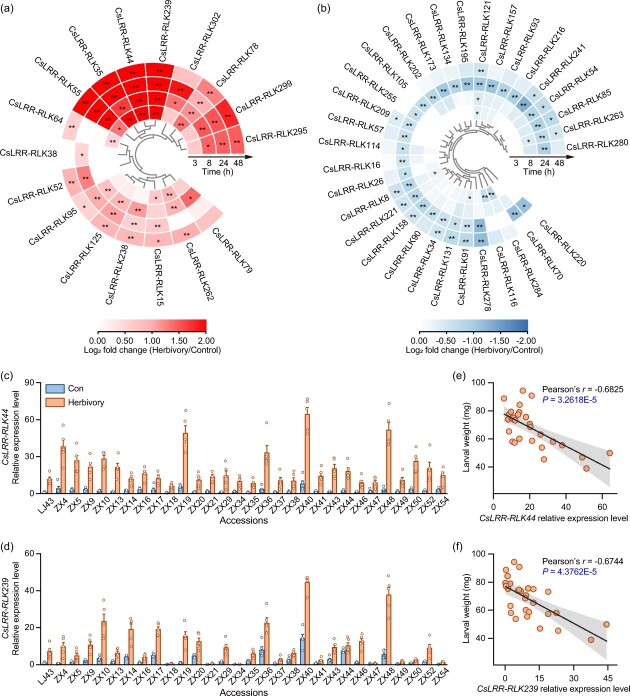
**Herbivory-induced expression of *CsLRR-RLK44* and *CsLRR-RLK239* is associated with tea resistance to herbivores. (a, b)** Heat maps displaying the expression of 17 upregulated *CsLRR-RLKs* (a) and 32 downregulated *CsLRR-RLKs* (b) under herbivory treatment. Color intensity reflects the log_2_-transformed fold changes in *CsLRR-RLK* expression in treated plants compared to control plants (*n* = 5). For the absolute expression values, refer to Table S8. Asterisks denote significant differences between treatments (evaluated using two-way ANOVA followed by pairwise comparisons with FDR-corrected LSMeans; ^*^P < 0.05; ^**^P < 0.01). **(c, d)** Transcript levels of *CsLRR-RLK44* (c) and *CsLRR-RLK239* (d) in different tea accessions before and after herbivory (+ SE, *n* = 5). Colored dots on each plot represent individual data points from replicates. **(e, f)** Correlations between herbivore-induced expression of *CsLRR-RLK44* (e) or *CsLRR-RLK239* (f), and larval weight gain. The Pearson’s product–moment correlation coefficient (*r*) and corresponding *P* values are provided. LJ43, Longjing 43.

To investigate the genetic diversity and functional traits of LRR-RLKs in tea plants, we carried out an analysis of the gene structure and conserved domains of *CsLRR-RLKs*. Detailed counts and distributions of introns and exons are provided in Table S4, with representative genes from each subgroup illustrated in Fig. 2b. Notably, nearly 40% of *CsLRR-RLKs* (120 out of 307) contained a single intron, while 7 genes lacked introns altogether. Additionally, 67 *CsLRR-RLKs* harbored more than 8 introns, with 30 of them containing >15 introns, exemplified by *CsLRR-RLK95* in Subgroup VIII-1 with 39 introns (Table S4).

Using DeepTMHMM, Pfam, and SMART, we analyzed the typical domains of CsLRR-RLK proteins, with a focus on the presence of signal peptides, the types and numbers of LRRs, transmembrane domains, kinase domain types, and other relevant features. For the extracellular LRRs, all members within each LRR-RLK subgroup contained multiple types of LRR domains, notably LRR_8, LRRNT_2, and LRR_4, with distinct distribution patterns in each subgroup (Fig. 2c, Tables S5 and S6). Regarding the intracellular kinase domains, there were mainly two types: the serine/threonine protein kinase (Ser/Thr kinase, S_TKc) and the serine/threonine/tyrosine protein kinase (Ser/Thr/Tyr kinase, STYKc), with different subfamilies showing preferences for different kinase domain types. For example, Subfamilies I, II, VIII-1, VIII-2, IX, XI, and XIII predominantly contained the Ser/Thr kinase domain, while Subfamilies III–VII, XII, and XIV were more inclined to have the Ser/Thr/Tyr kinase domain. Subfamily X members had both types (Fig. 2c). Signal peptide prediction showed that ~60% of CsLRR-RLKs (185 out of 307) contained a signal peptide. Furthermore, the transmembrane domain analysis revealed that CsLRR-RLKs with a single transmembrane domain were predominant (279 out of 307, >90%, Table S7). Additionally, members of Subfamilies VIII-2 and I exhibited characteristic domains such as Malectin and Malectin-like, respectively (Fig. 2c). These findings indicate that CsLRR-RLKs within the same group have similar structures, supporting the results of our phylogenetic analysis.

### *CsLRR-RLK44* and *CsLRR-RLK239* are involved in tea resistance to herbivores

To explore herbivore-related LRR-RLKs in tea plants, we performed a comparative transcriptome analysis of tea leaves before and after herbivory at different time points. Using a *P*-value threshold of <.05 to identify differentially expressed genes, we found that 49 *CsLRR-RLKs* exhibited significant changes in expression, with 17 genes showing remarkable upregulation and 32 genes displaying downregulation (Fig. 3a, b, Table S8). Notably, while more genes were downregulated than upregulated, the degree of upregulation was much higher than that of downregulation. Of the 17 upregulated genes, 13 showed at least a 2-fold increase in expression at one or more time points. Among the 32 downregulated genes, only eight exhibited a reduction in expression by >50%, and of these eight genes, seven showed significant reductions only at the 24-h time point (Table S8), indicating that herbivory has a much stronger inductive effect on *CsLRR-RLK* genes than a suppressive effect. We then focused on eight genes that clustered together and displayed the most pronounced upregulation: *CsLRR-RLK35*, *CsLRR-RLK44*, *CsLRR-RLK55*, *CsLRR-RLK78*, *CsLRR-RLK239*, *CsLRR-RLK295*, *CsLRR-RLK299*, and *CsLRR-RLK302* (Fig. 3a). Detailed quantitative real-time polymerase chain reaction (QRT-PCR) analysis confirmed that these eight *CsLRR-RLKs* responded strongly to tea geometrid attack, suggesting their potential involvement in herbivore-related defense responses (Fig. S1).

To identify the regulators of herbivore resistance among these eight *CsLRR-RLKs*, we measured their transcript levels in the leaves of 30 tea accessions after herbivore attack (Fig. 3c, d and Fig. S2). These accessions displayed significant variation in herbivore resistance [26]. We then analyzed the correlation between the larval growth of tea geometrid and expression levels of these eight *CsLRR-RLKs* individually. Notably, the herbivore-induced expression levels of *CsLRR-RLK44* and *CsLRR-RLK239*, but not the remaining six *CsLRR-RLK* genes, exhibited a negative correlation with herbivore growth (Fig. 3e, f and Fig. S2). These findings suggest that tea resistance to herbivores is associated with induced expression of *CsLRR-RLK44* and *CsLRR-RLK239*.

### *CsLRR-RLK44* and *CsLRR-RLK239* localize to the plasma membrane and respond strongly to herbivory

**Figure 4:**
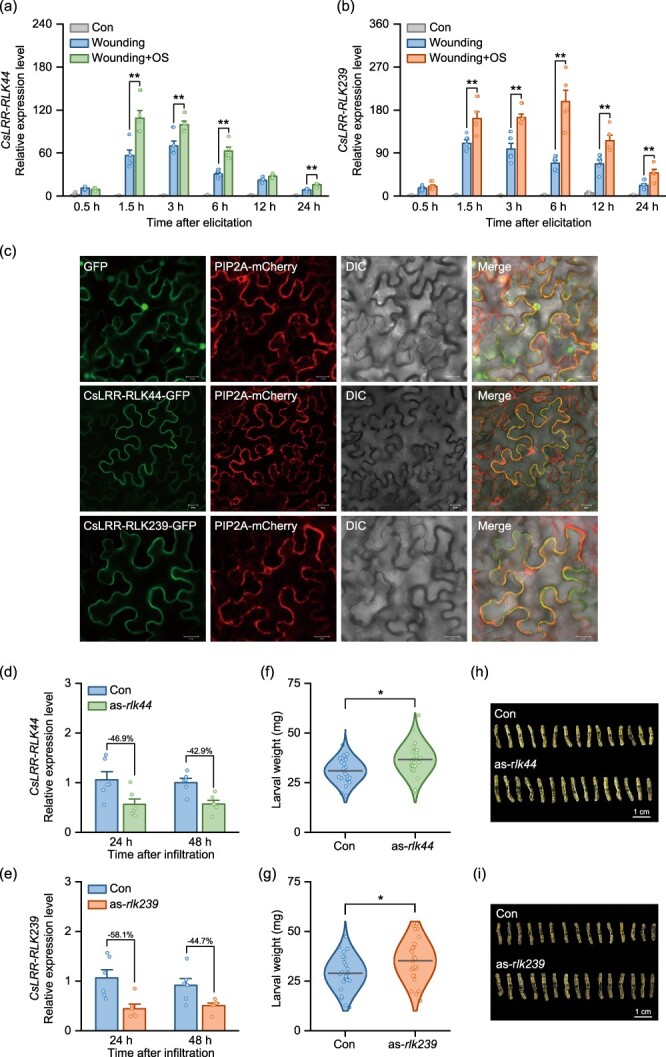
***CsLRR-RLK44* and *CsLRR-RLK239* positively regulate tea resistance to tea geometrid larvae. (a, b)** Transcript levels of *CsLRR-RLK44* and *CsLRR-RLK239* after treatment with mechanical wounding or simulated herbivory (wounding + oral secretion [OS]) (+ SE, *n* = 6). **(c)** Subcellular localizations of CsLRR-RLK44 and CsLRR-RLK239. Each *CsLRR-RLK44–* or *CsLRR-RLK239–GFP* fusion was transiently expressed in *N. benthamiana* leaf epidermal cells. Co-localization was observed with the Arabidopsis plasma membrane marker AtPIP2A–mCherry. Images displayed in sequence: GFP signal, plasma membrane stained with mCherry, differential interference contrast (DIC), and a composite overlay of all three signals. **(d, e)**  *CsLRR-RLK44* and *CsLRR-RLK239* expression levels in their respective silenced tea plants, as-*rlk44* (d) and as-*rlk239* (e) (+ SE, *n* = 6). Asterisks denote significant differences between treatments (evaluated using two-way ANOVA followed by pairwise comparisons with FDR-corrected LSMeans; ^*^P < 0.05; ^**^P < 0.01). **(f, g)** Larval weight gain of tea geometrid feeding on control (Con), *CsLRR-RLK44*-silenced (as-*rlk44*, f) or *CsLRR-RLK239*-silenced (as-*rlk239*, g) tea plants (*n* = 25). The horizontal line overlaid onto the violin plots represent the mean value of each treatment. Asterisks denote significant differences between treatments (evaluated using two-sided student’s *t-*test, ^*^P < 0.05). Colored dots on each plot represent individual data points from replicates. **(h, i)** Representatives of tea geometrid larvae at 6 days after feeding on Con or as-*rlk44* (h), or as-*rlk239* (i) plants.

To determine the specificity of *CsLRR-RLK44* and *CsLRR-RLK239* in response to herbivory, we quantified their expression after mechanical wounding and simulated herbivory (wounding + oral secretion). The results clearly showed that the application of oral secretions from tea geometrid to the wounds dramatically amplified the expression of these two RLKs, far exceeding the induction caused by mechanical wounding alone (Fig. 4a, b). Next, we investigated the subcellular localization of CsLRR-RLK44 and CsLRR-RLK239. The coding regions of these two CsLRR-RLKs were individually fused to green fluorescent protein (GFP) at their C-terminus and expressed in tobacco (*Nicotiana benthamiana*) leaves under the control of the CaMV 35S promoter (constructs: *35S::CsLRR-RLK44-GFP* and *35S::CsLRR-RLK239-GFP*). Consistent with the membrane-localized marker AtPIP2A [28], both CsLRR-RLK44-GFP and CsLRR-RLK239-GFP exhibited a fluorescent signal distinctly localized to the plasma membrane (Fig. 4c). Taken together, these findings suggest that *CsLRR-RLK44* and *CsLRR-RLK239* respond strongly to HAMPs and may function as components of a plasma membrane-localized receptor system.

### *CsLRR-RLK44* and *CsLRR-RLK239* do not function as homodimers or heterodimers

Previous studies have shown that LRR-RLKs may fully function by forming homodimers with themselves or heterodimers with other LRR-RLKs [29, 30]. To investigate whether CsLRR-RLK44 and CsLRR-RLK239 form such dimers, we performed yeast two-hybrid (Y2H) and bimolecular fluorescence complementation (BiFC) assays. Our results indicate no interaction between CsLRR-RLK44 and CsLRR-RLK239, nor did either protein interacted with itself (Fig. S3). These findings suggest that CsLRR-RLK44 and CsLRR-RLK239 are unlikely to form heterodimers with each other or homodimers.

### Silencing *CsLRR-RLK44* and *CsLRR-RLK239* decreases herbivore resistance

To further elucidate the role of *CsLRR-RLK44* and *CsLRR-RLK239* in herbivore resistance in tea plants, we transiently suppressed their expression in tea leaves with an antisense oligodeoxynucleotide (asODN) approach [31], as stable transformation methods for tea plants are current unavailable. QRT-PCR analysis revealed a significant reduction in the expression levels of *CsLRR-RLK44* or *CsLRR-RLK239* in tea leaves treated with their respective asODNs. This suppression effect persisted for at least 48 h post-infiltration, with a silencing efficiency of ~50% for both genes at this time point, underscoring the efficacy of the asODN method for both genes in tea plants (Fig. 4d, e). We also examined the expression levels of *CsLRR-RLK178* and *CsLRR-RLK238*, the most homologous to *CsLRR-RLK44* and *CsLRR-RLK239*, respectively, and found that their expression was unaffected, indicating high specificity of the silencing method (Fig. S4). The tea geometrid larvae fed on *CsLRR-RLK44* or *CsLRR-RLK239* suppression plants (as-*rlk44* or as-*rlk239*) exhibited accelerated growth compared to those fed on control plants (Fig. 4f–i). Thus, *CsLRR-RLK44* and *CsLRR-RLK239* play critical roles in enhancing herbivore resistance in tea plants.

### *CsLRR-RLK44* and *CsLRR-RLK239* regulate defense-related MPKs and WRKYs

**Figure 5:**
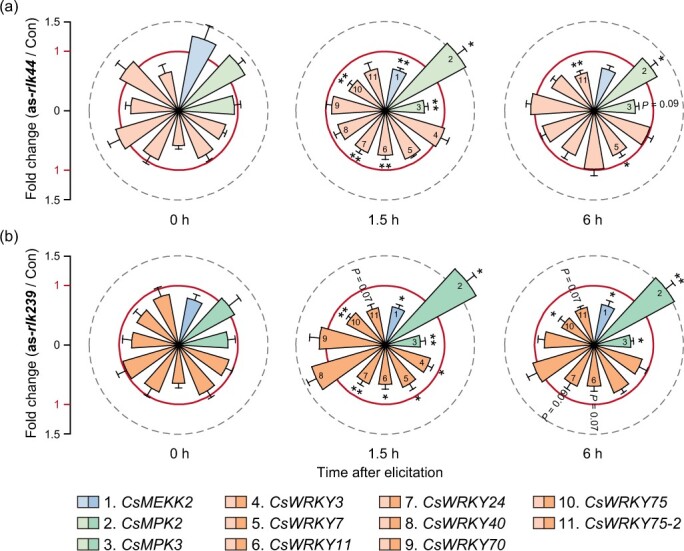
***CsLRR-RLK44* and *CsLRR-RLK239* regulate defense-related MPKs and WRKYs expression.** Fold changes in the expression of *MEKK*, *MPK*, and *WRKY* genes in **(a)**  *CsLRR-RLK44*-silenced or **(b)**  *CsLRR-RLK239*-silenced (as-*rlk44* or as-*rlk239*) tea plants relative to control tea plants at different time points after herbivory (+ SE, *n* = 6). Absolute values are detailed in Figs. S5–S7. Asterisks denote significant differences between treatments (evaluated using two-way ANOVA followed by pairwise comparisons with FDR-corrected LSMeans; ^*^P < 0.05; ^**^P < 0.01).

Some LRR-RLKs initiate early defenses via activating MPK cascades [32]. To determine whether *CsLRR-RLK44* and *CsLRR-RLK239* have the capacity to regulate MPK signaling, we profiled the expression of *CsMEKK20*, *CsMPK2*, and *CsMPK3* after silencing *CsLRR-RLK44* or *CsLRR-RLK239*. *CsMEKK20* belongs to the MAPKKK family, and was significantly induced after simulated tea geometrid feeding (Fig. S5A). *CsMEKK20* is the ortholog of *AtMEKK20* in Arabidopsis. AtMEKK20 is a key component that phosphorates MPKs and thereby activates plant immunity [33]. *CsMPK2* and *CsMPK3* have been implicated in the defense response against tea geometrid [34]. In silenced plants (as-*rlk44* and as-*rlk239*), we observed a significant decrease in the expression of *CsMEKK20* and *CsMPK3* compared to control plants after herbivory elicitation, whereas *CsMPK2* was increased (Fig. 5, Fig. S5). These results show that CsLRR-RLK44 and CsLRR-RLK239 play a role in modulating MPK signaling.

LRR-RLKs can regulate WRKY transcription factors, which subsequently modulate plant defense responses [35]. To delve deeper into how *CsLRR-RLK44* and *CsLRR-RLK239* regulate early defense signaling, we examined the expression of eight WRKYs in as-*rlk44* and as-*rlk239* plants. These WRKYs have been demonstrated to play roles in both biotic and abiotic stresses in tea plants [36–38]. Silencing *CsLRR-RLK44* reduced the expression levels of herbivore-induced *CsWRKY7*, *CsWRKY11*, *CsWRKY24*, *CsWRKY75*, and *CsWRKY75–2*, whereas it did not affect *CsWRKY3*, *CsWRKY40*, and *CsWRKY70* expression (Fig. 5a and Fig. S6). Similarly, silencing *CsLRR-RLK239* reduced the expression levels of *CsWRKY3*, *CsWRKY7*, *CsWRKY11*, *CsWRKY24*, and *CsWRKY75*, and *CsWRKY75–2* displayed a decreased tendency (Fig. 5b and Fig. S7). Thus, *CsLRR-RLK44* and *CsLRR-RLK239* specifically regulate a subset of defense-related WRKY transcription factors.

### Silencing *CsLRR-RLK44* and *CsLRR-RLK239* decreases herbivore-elicited jasmonate biosynthesis

**Figure 6:**
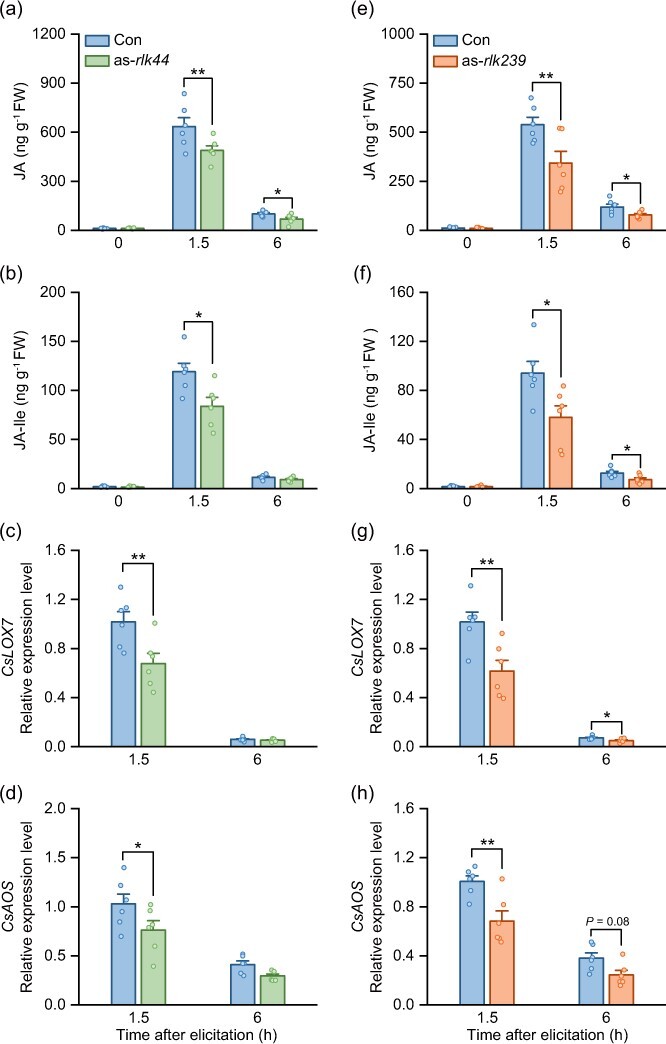
***CsLRR-RLK44* and *CsLRR-RLK239* positively regulates herbivore-elicited jasmonate biosynthesis. (a, b)** Levels of JA and JA-Ile in *CsLRR-RLK44*-silenced (as-*rlk44*) tea plants after herbivory elicitation (+SE, *n* = 6). **(c, d)** Expression levels of jasmonate biosynthesis-related gene *CsLOX7* and *CsAOS* in as-*rlk44* plants after herbivory elicitation (+SE, *n* = 6). **(e, f)** Levels of JA and JA-Ile in *CsLRR-RLK239*-silenced (as-*rlk239*) tea plants after herbivory elicitation (+SE, *n* = 6). **(g, h)** Expression levels of jasmonate biosynthesis-related gene *CsLOX7* and *CsAOS* in as*-rlk239* plants after herbivory (+SE, *n* = 6). Colored dots on each plot represent individual data points from replicates. Asterisks denote significant differences between treatments (evaluated using two-way ANOVA followed by pairwise comparisons with FDR-corrected LSMeans; ^*^P < 0.05; ^**^P < 0.01).

Jasmonate is the core signaling that triggers plant defenses against chewing herbivores [39–41]. *CsLOX7* and *CsAOS*, key genes in the JA biosynthesis pathway, have been shown to be particularly closely associated with resistance to the tea geometrid in tea plants, with their expression patterns mirroring the levels and dynamics of JA induced by herbivory [26, 34, 42, 43]. To explore whether early defense signaling regulation is linked to changes in jasmonate signaling, we first measured the herbivore-induced JA and JA-Ile contents in tea plants after silencing *CsLRR-RLK44* or *CsLRR-RLK239*, respectively. We found that silencing of *CsLRR-RLK44* or *CsLRR-RLK239* significantly decreased JA and JA-Ile levels at 1.5 and 6 h after herbivory (Fig. 6). Additionally, transcript levels of *CsLOX7* and *CsAOS* were notably lower in silenced plants (as-*rlk44* and as-*rlk239*) compared to controls (Fig. 6). Together, these results demonstrate that *CsLRR-RLK44* and *CsLRR-RLK239* play a positive role in herbivory-induced jasmonate biosynthesis.

## Discussion

LRR-RLKs represent one of the largest families of cell surface receptors implicated in initiating plant defense signaling. While their functions in grass plants such as Arabidopsis, rice, and soybean have been gradually elucidated, the roles of herbivory-related LRR-RLKs in woody plants are largely unknown. Our study addresses this knowledge gap by identifying and characterizing two CsLRR-RLKs essential for activating defense signaling and enhancing herbivore resistance in tea plants.

Through genome mining of tea plants, we identified a total of 307 intact LRR-RLKs, among which 49 *CsLRR-RLKs* were regulated by herbivore infestation. Focusing on the eight most prominently upregulated RLKs, we observed that only the expression of *CsLRR-RLK44* and *CsLRR-RLK239* exhibited a significant correlation with herbivore resistance across different tea accessions. Phylogenetic and structural analyses revealed that both *CsLRR-RLK44* and *CsLRR-RLK239* belong to the XI subgroup. Evolutionary tree and homology comparison analyses further indicated that CsLRR-RLK44 is most closely related to *A. thaliana* AtRLK7 (AT1G09970) (Fig. S8), which is known to regulate tolerance to oxidative stress and *Fusarium graminearum* infection [44, 45]. Additionally, AtRLK7 initiates plant immune signaling after perception of PAMP-induced secreted peptides (PIPs) [46]. Similarly, CsLRR-RLK239 is clustered with AtHSL3 (AT5G25930) (Fig. S9), involved in immunity to pathogens and herbivores [47]. These findings indicate that *CsLRR-RLK44* and *CsLRR-RLK239* likely participate in herbivore-induced defense responses.

As membrane-localized pattern recognition receptors, AtRLK7 has been shown to perceive peptides such as PIP1, PIP2, PIP3, PIPL6, and TOLS2, triggering distinct responses [46, 48–51]. AtHSL3 is induced by pathogens and binds to the secreted peptides like ‘small phytocytokines regulating defense and water loss’ (SCREWs) and CTNIP [47, 52]. Similar to AtRLK7 and AtHSL3, both CsLRR-RLK44 and CsLRR-RLK239 localize to the plasma membrane. They exhibited low expression levels in non-treated plants but were rapidly induced upon herbivore attack. Furthermore, their induction by larval oral secretions was significantly stronger compared to mechanical wounding alone, indicating the specificity of their response to herbivory. Given the ability of AtRLK7 and AtHSL3 to bind various peptides induced by biotic and abiotic stresses, it is conceivable that CsLRR-RLK44 and CsLRR-RLK239 may perceive herbivory-associated signaling elements, such as DAMPs or HAMPs, or be indirectly induced by other herbivory-related components, such as plant peptides, thereby eliciting downstream defense responses. Nevertheless, more extensive and in-depth research is needed to validate this hypothesis. The identification of ligands or interacting components of CsLRR-RLK44 and CsLRR-RLK239 holds promise for expanding our limited understanding of how herbivory activates plant defense systems. While some LRR-RLKs fully function by forming homodimers with themselves or heterodimers with other LRR-RLKs [29, 30], our Y2H and BiFC experiments showed that neither CsLRR-RLK44 nor CsLRR-RLK239 forms heterodimers with each other or homodimers with themselves. However, we cannot rule out the possibility that these proteins may interact with other, as-yet unidentified LRR-RLKs to form dimers. This potential interaction warrants further exploration in future studies. Our study provides valuable insights into candidate receptors involved in herbivory perception and the initiation of plant defenses.

Certain LRR-RLKs have been identified as initiators of plant defenses through MPK signaling. For instance, in rice, *OsLRR-RLK1* positively regulates the herbivore-elicited expression of *OsMEK4*, *OsMPK3*, and activates OsMPK3 and OsMPK6, while *OsLRR-RLK2* promotes the expression of *OsMPK6* and activates OsMPK3 after herbivore attack [15, 20]. Similarly, under different circumstances, AtRLK7 activates AtMPK3/MPK6 for signal amplification [46, 48, 50, 51]. In our study, *CsLRR-RLK44* and *CsLRR-RLK239* demonstrated the capacity to regulate MPK signaling, as evidenced by the altered expression of *CsMEKK20* and *CsMPK3* in *CsLRR-RLK44*- or *CsLRR-RLK239*-silenced plants. Nevertheless, it’s important to emphasize that as protein kinases, MPKs rely on the phosphorylation process to amplify upstream signaling and transduce the amplified signaling to downstream targets such as transcription factors. Therefore, it is crucial to determine whether *CsLRR-RLK44* and *CsLRR-RLK239* influence the activation status of MPKs, which is a gap in the current study. Additionally, the specificity of *CsLRR-RLK44* and *CsLRR-RLK239* in regulating MPKs, including the observed increase in *CsMPK2*, suggests a unique regulatory mechanism that remains to be fully understood. To date, some complete defense-related MPK cascades have been characterized under the control of LRR-RLKs in grass plants, for instance, CERK1–PBL27–MAPKKK5–MKK4/MKK5–MPK3/MPK6 in Arabidopsis and OsCERK1–OsRLCK185–OsMAPKKK18–OsMKK4–OsMPK3/OsMPK6 in rice [53]. Exploring the exact cascades of LRR-RLKs activating their downstream MPKs in woody plants after herbivory presents an exciting prospect for future research.

Here, we discovered that *CsLRR-RLK44* and *CsLRR-RLK239* regulate a subset of defense-related WRKY transcription factors, including *CsWRKY3*, *CsWRKY7*, *CsWRKY11*, *CsWRKY24*, *CsWRKY75*, and *CsWRKY75–2*. Notably, *CsWRKY3* has been reported to trigger resistance against the pathogen *Colletotrichum fructicola* and the herbivore *Empoasca onukii* in tea plants [36]. MPKs are well-known upstream regulators of WRKY transcription factors. For instance, in tobacco, the ortholog of *CsWRKY3*, *NtWRKY3*, is transcriptionally regulated by MPKs such as *WIPK* and *SIPK* to propagate downstream defense signaling [54, 55]. These MPKs directly phosphorylate the SP cluster of WRKY transcription factors [56]. Similarly, in rice, the orthologs of *CsWRKY24* and *CsWRKY70*, known as *OsWRKY24* and *OsWRKY70*, are not only physically interacted with and phosphorylated by *OsMPK3* and *OsMPK6*, but also regulated at the transcriptional level [57]. Besides, our *CsMPK3* is also the ortholog of *WIPK* and *OsMPK3*. These findings strongly suggest that the modulation of defense-related WRKYs likely involves the MPK cascade, which is influenced by *CsLRR-RLK44* and *CsLRR-RLK239*.

*CsLRR-RLK44* and *CsLRR-RLK239* were observed to enhance the herbivory-induced levels of JA and JA-Ile, along with increasing the expression of JA biosynthesis genes, *CsLOX7* and *CsAOS*. Suppression of *CsLRR-RLK44* and *CsLRR-RLK239* resulted in decreased resistance to tea geometrid larvae. Given that jasmonate is a well-known core signaling molecule in triggering plant resistance to chewing herbivores, and emerging evidence indicates that LRR-RLKs such as OsLRR-RLK1 and AtPEPR1/PEPR2 convey herbivore resistance via JA signaling, it is thus unsurprising that *CsLRR-RLK44* and *CsLRR-RLK239* may regulate tea resistance to tea geometrid by enhancing the jasmonate signaling. Furthermore, comprehensive research has demonstrated that MPKs and WRKYs play crucial roles in the biosynthesis of herbivore-induced JA. For instance, OsMPK3 positively modulates herbivory-elicited JA levels via the activation of OsWRKY70 and OsWRKY53 [57, 58]. Given their strong influences on MPKs and WRKYs found here, it is plausible that *CsLRR-RLK44* and *CsLRR-RLK239* may regulate JA biosynthesis through MPK and WRKY signaling. However, additional experiments employing silencing plants of the cascade genes such as as-*mpk3*, as-*wrky3*, and as-*wrky24* would be necessary to validate this hypothesis. Additionally, we observed that *CsLRR-RLK44* and *CsLRR-RLK239* did not regulate MPKs, WRKYs, and jasmonates at 0 h. Instead, these genes were regulated following herbivory. The expression of *CsLRR-RLK44* and *CsLRR-RLK239* was strongly induced by herbivory, and their associated MPKs and WRKYs were also significantly upregulated only after herbivory. This suggests that the regulatory role of these two *CsLRR-RLKs* is activated post-herbivory.

## Conclusion

**Figure 7:**
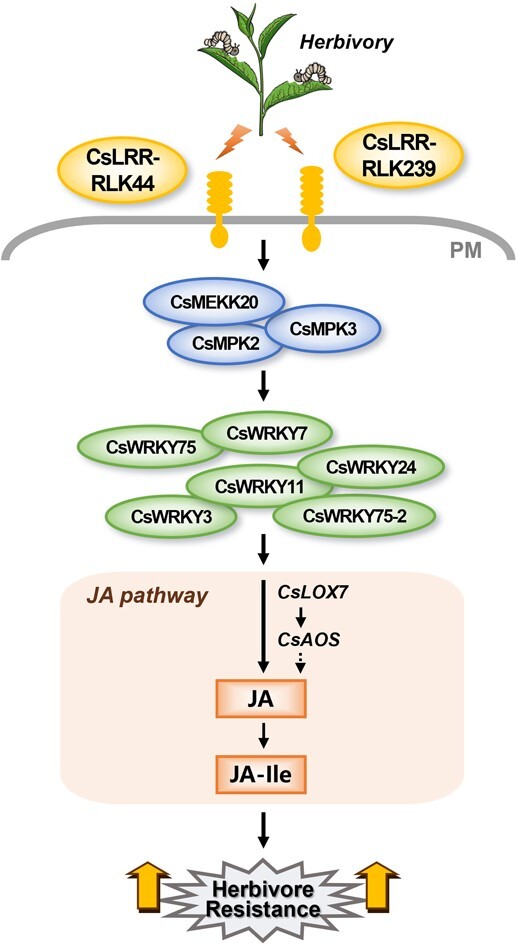
**Proposed model for *CsLRR-RLKs* in regulating herbivore-induced signaling pathways and defenses in tea plants.** After herbivory, *CsLRR-RLK44* and *CsLRR-RLK239* are rapidly and strongly induced. These two *LRR-RLKs* modulate the expression of downstream MPK cascades and specific WRKY transcription factors. The changed expression of MPKs and WRKYs may modulate JA biosynthesis genes such as *CsLOX7* and *CsAOS*, resulting in increased jasmonates, which consequently enhance tea defenses against tea geometrid larvae. PM, plasma membrane.

In summary, we propose the following model. Upon herbivory, *CsLRR-RLK44* and *CsLRR-RLK239* are rapidly and strongly induced. These plasma membrane-localized CsLRR-RLKs may either perceive HAMPs or be regulated by other herbivory-related signaling molecules. Subsequently, CsLRR-RLK44 and CsLRR-RLK239 enhance the expression of MPKs, which may modulate the expression of WRKYs. These altered expression of MPKs and WRKYs then modulate the biosynthesis of herbivore-elicited JA, thereby bolstering defenses against tea geometrid (Fig. 7). Our study is the first to demonstrate that in woody plants, LRR-RLKs are also essential for regulating herbivore resistance through the activation of the canonical signaling pathway, including MPKs, WRKYs, and JA pathway. Furthermore, our work extends mechanistic insights on LRR-RLKs initiating plant defenses from grasses to economically important tree species.

## Materials and methods

### Comprehensive identification and characterization of *CsLRR-RLKs*

To comprehensively identify and characterize the LRR-RLK genes in tea plants, we began by retrieving LRR-RLK protein sequences from the Arabidopsis genome database (The Arabidopsis Information Resource). Using these sequences as queries, we employed the BLASTP program (E-value <10^−5^) to search the protein database of the tea cultivar Longjing 43 (LJ43) [59]. In addition, we downloaded the Hidden Markov Model (HMM) profiles of LRR domains [LRR_1 (PF12799), LRRNT_2 (PF08263), LRR6 (PF13516), LRR8 (PF13855)] and protein kinase domains (PF00069, PF07714) from the Pfam v32.0 database [60]. These profiles were used to screen the tea proteome sequences via the HMMER program with an E-value cut-off of 10^−5^. All resulting candidates were further confirmed via eliminating the redundant sequences and functional annotations and then analysis with the online tool NCBI CD-Search [61] and EMBL SMART [62]. The presence of transmembrane domains was assessed with DeepTMHMM [63]. Sequences lacking LRR motifs, RLK domains, or transmembrane domains were manually excluded. The remaining candidate genes, containing at least one LRR motif, a transmembrane domain, and a protein kinase domain, were designated as putative CsLRR-RLKs and named according to their chromosome or scaffold locations. We used the online tool ProtParam to predict the physicochemical parameters of CsLRR-RLK proteins, including molecular weight, amino acid length, as well as theoretical isoelectric point [64].

To examine the distribution of *CsLRR-RLKs* across LJ43 chromosomes, we downloaded genomic sequences and genome annotation files of LJ43 from the Genome Sequence Archive in the Beijing Institute of Genomics (BIG) database (accession number PRJCA001158) [59]. The chromosomal locations of *CsLRR-RLKs* were then illustrated using MG2C v2.1 online tool.

### Phylogenetic analysis of CsLRR-RLKs

To further elucidate the phylogenetic relationship of CsLRR-RLKs and aid their classification, multiple sequence alignment of CsLRR-RLKs and AtLRR-RLKs was first carried out using BioEdit v7.0.9 software with default settings [65]. A phylogenetic tree of CsLRR-RLKs and AtLRR-RLKs was then constructed based on the conserved LRR and protein kinase domain sequences. This was done using the Neighbor-Joining method with MEGA 7.0 [66] employing 1000 bootstrap replicates, the Poisson model, and pairwise deletion for statistical support. The online program iTOL [67] was then used to visualize and improve graphical representations of the tree.

### Gene structure and conserved domain analysis of CsLRR-RLKs

To characterize the structural divergence of *CsLRR-RLKs* in each subgroup, we obtained the coding sequences and genome annotation information of *CsLRR-RLK* gene family from BIG database (accession number PRJCA001158) [59]. The online tool GSDS 2.0 was employed to generate the exon–intron structure diagram for the gene family [68]. We used DeepTMHMM [63] to analyze signal peptides and transmembrane domains, while SMART [62] and Pfam [60] were employed to identify LRR motifs, protein kinase domains, and other characteristic domains commonly found in LRR-RLKs. The results, including the gene structure diagram and conserved domains of several representative proteins from each subfamily, were consolidated and displayed using TBtools software [69].

### Plants and herbivores

To explore the role of *CsLRR-RLKs* in herbivore resistance, we utilized 30 tea accessions previously described and characterized, including their resistance to tea geometrid (*E. obliqua*) larvae [26]. For transcriptome analysis, gene expression studies, gene silencing, herbivore performance assessment, and phytohormone quantification, we used 3-year-old LJ43 tea plants. These plants were grown in pots with commercial potting soil and maintained in a greenhouse environment (24°C ± 2°C, 70%–80% relative humidity, 14, 10 h light/dark cycle). Healthy, uniformly sized tea plants were selected and allowed to recover before further experimentation, as previously described [34, 70]. Tea geometrid larvae (*E. obliqua*) were collected from a natural tea plantation and reared in a climate chamber set at 26°C, 70%–80% relative humidity, and a 12:12-h photoperiod. Oral secretions were collected from forth-instar *E. obliqua* larvae that had been feeding on tea leaves and stored in −80°C before use.

### Herbivory treatment

For herbivore infestation, we placed two third-instar tea geometrid larvae on the second fully expanded leaves and enclosed them in small mesh pockets (9 × 11 cm) to prevent escape. Control leaves were covered with empty mesh pockets. Whole leaves were collected at designated time intervals after herbivore elicitation.

To determine the specificity of *CsLRR-RLKs* in response to herbivory, tea leaves were subjected to mechanical wounding and then treated with oral secretions to simulate herbivory, as previously described [34]. Leaves only challenged by mechanical wounding or without any treatment were set as controls. Whole leaves were collected at designated time points after the treatments.

### Transcriptome analysis

Leaves of tea plants, infested with tea geometrid larvae for 3, 8, 24, and 48 h, were collected following the above outlined procedure. Non-infested healthy tea leaves at corresponding time points were served as controls. Each treatment involved pooling four leaves together to constitute one biological replicate, with five biological replicates performed for each treatment. Total RNA was extracted with the RNAprep Pure Plant Plus Kit (TIANGEN, China) and assessed for integrity with the RNA Nano 6000 Assay Kit (Agilent Technologies, USA). Subsequently, high-quality RNA samples were utilized for constructing cDNA libraries and sequencing on the Illumina NovaSeq 6000 platform at Novogene Bioinformatic Technology Co., Ltd. (Tianjing, China). The raw sequencing data underwent initial processing using custom Perl scripts to eliminate adapter contaminants and low-quality reads, yielding high-quality clean data. An index of the LJ43 reference genome was created, and paired-end clean reads were aligned to this reference using Hisat2 v2.0.5 [71]. FeatureCounts vl.5.0 [72] was employed to count the reads mapped to individual genes, followed by the calculation of FPKM (Fragments per kilobase of transcript per million mapped reads) for each gene. Differential expression analysis between control and herbivory treatments was conducted using the DESeq2 v1.20.0 R package. Genes showing a resulting *P-*value <.05 were designated as differentially expressed.

### Quantitative real time-PCR

Gene expression analysis was conducted using QRT-PCR to quantify transcript levels of target genes. Briefly, total RNA was extracted from tea leaves with RNAprep Pure Plant Plus Kit (TIANGEN, China). Each sample underwent reverse transcription of 500 ng of total RNA using the PrimerScriptTM RT Master Mix (Takara, China). QRT-PCR was conducted on the LightCycler 384 System (Roche Diagnostics GmbH, Germany) with the SYBR Green I Master, following an initial denaturation at 95°C for 4 min, followed by 40 cycles of amplification (95°C for 10 s, 60°C for 20 s). *CsGAPDH* served as the internal reference control. Specific primers employed for QRT-PCR analysis of all tested genes can be found in Table S9. Relative gene expression levels were determined using the 2^-ΔΔCt^ method, based on the threshold cycle (Ct) values [73].

### Isolation and subcellular localization of CsLRR-RLK44 and CsLRR-RLK239

The open reading frames (ORFs) of *CsLRR-RLK44* and *CsLRR-RLK239* were amplified using specific primers listed in Table S9. Following amplification, PCR products were cloned into pEASY-blunt zero vector (TransGen Biotech, China) and sequenced for confirm accuracy. Subsequently, the confirmed ORF sequences of *CsLRR-RLK44* and *CsLRR-RLK239*, excluding the termination codon, were then individually inserted into the pBI121 vector that contains the GFP reporter gene, resulting in *pBI121-CsLRR-RLK-GFP* fusion constructs. The vector *pBIN-AtPIP2A-mCherry* served as a positive control for membrane localization [28]. The plasmids were then transformed into *Agrobacterium tumefaciens* strain GV3101 via chemical transformation. Co-infiltration of *Agrobacterium* suspensions containing transformed *CsLRR-RLK-GFP* and the positive control vector *pBIN-AtPIP2A-mCherry* was conducted into leaves of 4-week-old *N. benthamiana* plants, using an optical density of 1.0 at 600 nm for transient expression [74]. Two days post-infiltration, small living sections from the infected areas of tobacco leaves were examined for fluorescence. GFP and mCherry-fusion proteins were excited at wavelengths of 488/507 nm and 587/610 nm, respectively, using a Zeiss LSM880 inverted confocal microscope (Carl Zeiss, Germany). Images were captured at a resolution of 1024 × 1024 pixels with software ZEN (Carl Zeiss).

### Silencing of *CsLRR-RLK44* and *CsLRR-RLK239* in tea plants

To elucidate the functional roles of *CsLRR-RLK44* and *CsLRR-RLK239* in herbivore resistance, asODN assays were performed to transiently silence these target gene as previously described [75, 76]. Candidate asODN sequences were obtained from the online software Soligo [77] with *CsLRR-RLK44* or *CsLRR-RLK239* as input sequences and synthesized by Zhejiang Youkang Biotechnology Company. The specific asODN sequences used are listed in Table S9. For gene silencing, 1 ml of 30 μM asODN solution was injected into the second fully expended leaves of tea plant seedlings. Leaves injected with sense oligonucleotides served as controls. After 24 and 48 h of incubation, the leaves underwent simulated herbivory treatment for 3 h and then harvested to assess the gene silencing efficiency and specificity. Additionally, the asODN-treated leaves were exposed to simulated herbivory treatment for 0, 1.5, and 6 h, then harvested for defense-related gene expression analysis and phytohormone quantification.

### Herbivore performance

Two tea geometrid larvae, 3 days old and of comparable size, were selected and allowed to feed on either control or *CsLRR-RLK-*silenced tea plants. To prevent the larvae from escaping, they were confined to the leaves of each plant using small mesh pockets measuring 9 × 11 cm. To ensure effective gene silencing and provide a sufficient food source, the leaves were replaced at least 48 h after the asODN treatment. Larval weight was recorded, and photographs were taken 6 days after initiating the experiment.

### Phytohormone quantification

To investigate the impact of *CsLRR-RLK44* and *CsLRR-RLK239* on herbivore-related phytohormone biosynthesis, we analyzed the concentrations of JA and JA-Ile in *CsLRR-RLK44-* or *CsLRR-RLK239-*silenced tea leaves. JA and JA-Ile were extracted using ethyl acetate spiked with isotopically labeled standards (1 ng each for d_6_-JA and d_6_-JA-Ile) and quantified using an ultra-high performance liquid chromatography-triple quadrupole mass spectrometry system (UPLC-MS/MS) following previously established protocols [34, 78].

### Yeast two-hybrid assay

The coding sequences of *CsLRR-RLK44* and *CsLRR-RLK239* were individually cloned into pGADT7 (AD) and pGBKT7 (BD) vectors. The appropriate combinations of recombinant plasmids were co-transformed into yeast strain AH109, according to the manufacturer’s instructions (Shanghai Weidi Biotechnology Co., Ltd, China). Transformed yeast cells were plated onto synthetic defined medium lacking Leu and Trp (SD-LW) and incubated at 30°C. After 3 days, positive yeast colonies were transferred to SD medium lacking Ade, His, Leu, and Trp (SD-LWAH) to confirm potential protein–protein interactions.

### Bimolecular fluorescence complementation

The coding sequences of *CsLRR-RLK44* and *CsLRR-RLK239* were individually inserted into the p2YN and p2YC vectors, which contain the N-terminal or C-terminal of the yellow fluorescent protein (YFP) reporter gene, respectively, resulting in *p2YN-CsLRR-RLKs* or *p2YC-CsLRR-RLKs* fusion constructs. The vectors *p2YN-CsSWEET1a* and *p2YC-CsSWEET1a* were used as a positive control pair for membrane-localized protein–protein interaction [79]. The plasmids were then transformed into *A. tumefaciens* strain GV3101. Co-infiltration of *Agrobacterium* suspensions containing the appropriate combinations of *p2YN-CsLRR-RLKs* or *p2YC-CsLRR-RLKs* constructs was carried out in the leaves of 4-week-old *N. benthamiana* plants. After 3 days of incubation, small living sections from the infiltrated areas of tobacco leaves were examined for fluorescence using a Zeiss LSM880 inverted confocal microscope (Carl Zeiss, Germany).

### Data analysis

We used one-way or two-way analysis of variance (ANOVA) to assess differences in herbivore performance, phytohormone levels, and gene expression across different treatments. Subsequently, pairwise comparisons were conducted using False Discovery Rate (FDR)-corrected Least Squares Means, as outlined in previous studies [80]. Correlations between *CsLRR-RLK* expression levels and herbivore growth were analyzed using the ‘cor.test’ function in R [81]. All statistical analyses were conducted using R 4.3.3 software (R Foundation for Statistical Computing). The number of independent biological replicates for each experiment is specified in the corresponding figure and figure legend.

## Supplementary Material

Web_Material_uhae281

## Data Availability

The data that support the findings of this study are available in the article and the Supplementary Information.
