# Supplementary material for: Two leucine-rich repeat receptor-like kinases initiate herbivory defense responses in tea plants
Source: Hortic Res. 2024 Oct 2;12(1):uhae281. doi: 10.1093/hr/uhae281 (PMC11756293; doi:10.1093/hr/uhae281)
Supplement: Web_Material_uhae281 [file web_material_uhae281.zip › CsLRR_Supplementary information.docx]

**Supplementary Information for**

**Two leucine-rich repeat receptor-like kinases initiate herbivory defense responses in tea plants**

Qi Jiang^†^, Changqing Ding^†^, Lingjia Feng, Zhenwei Wu, Yujie Liu, Lintong He, Chuande Liu, Lu Wang, Jianming Zeng, Jianyan Huang*, Meng Ye*

Key Laboratory of Biology, Genetics and Breeding of Special Economic Animals and Plants, Ministry of Agriculture and Rural Affairs, National Center for Tea Plant Improvement, Tea Research Institute, Chinese Academy of Agricultural Sciences, Hangzhou, China, 310008

*Correspondence: Meng Ye (yemeng@caas.cn) and Jianyan Huang ([jyhuang@tricaas.com](mailto:jyhuang@tricaas.com))

^†^These authors contributed equally to this work.


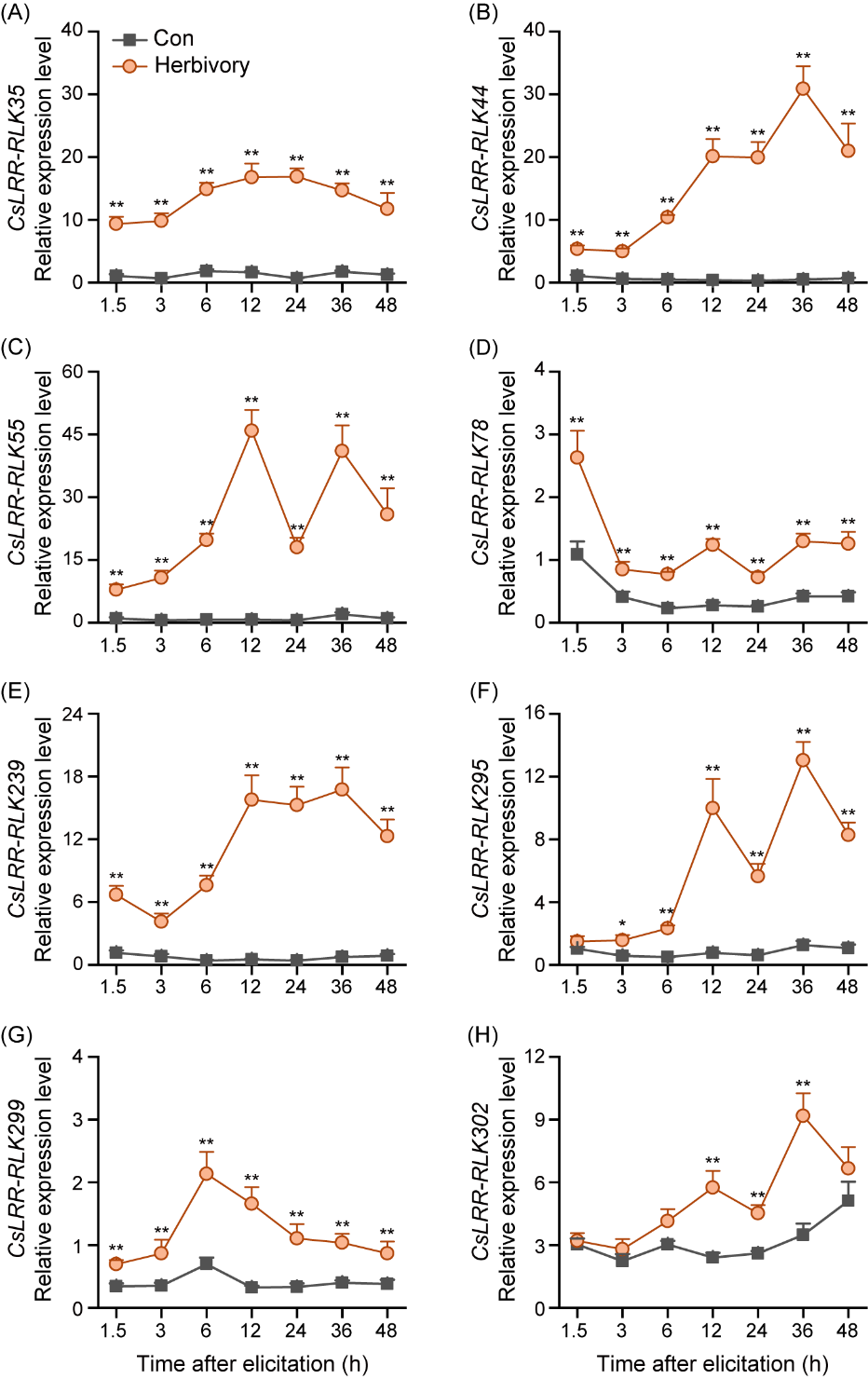


**Figure S1. Expression patterns of eight CsLRR-RLKs after herbivory.** The expression levels of *CsLRR-RLK35* **(A)**, *CsLRR-RLK44* **(B)**, *CsLRR-RLK55* **(C)**, *CsLRR-RLK78* **(D)**, *CsLRR-RLK239* **(E)**, *CsLRR-RLK295* **(F)**, *CsLRR-RLK299* **(G)** and *CsLRR-RLK302* **(H)** under tea geometrid attack (+ SE, *n* = 5-6). Con, control. Asterisks indicate significant differences between treatments at the same time points (two-way ANOVA followed by pairwise comparisons through FDR-corrected LSMeans; **P* < 0.05; ***P* < 0.01).


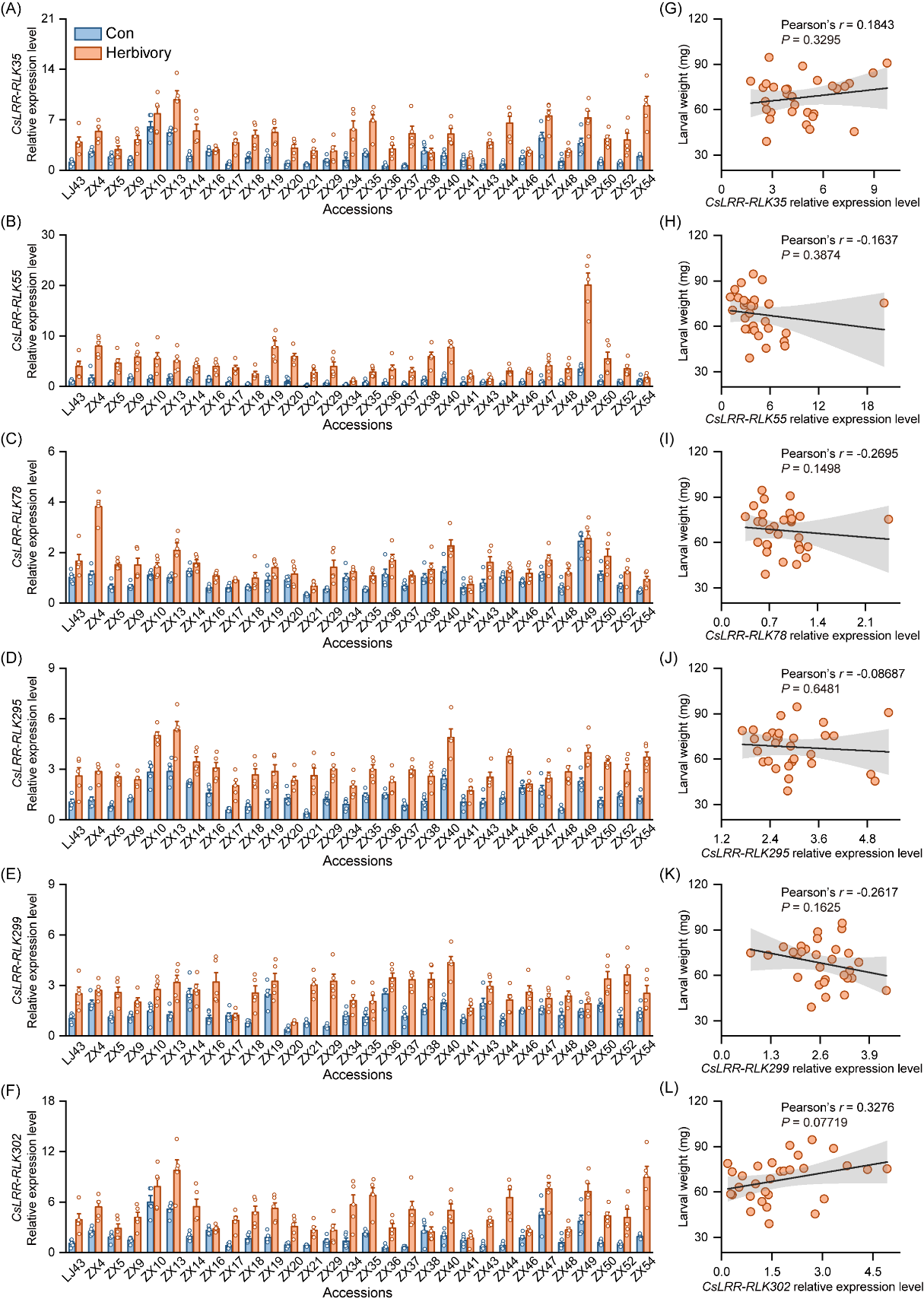


**Figure S2. Expression of *CsLRR-RLKs* and their correlations with herbivore growth in tea plants. (A-F)** Transcript levels of *CsLRR-RLK35* (A), *CsLRR-RLK55* (B), *CsLRR-RLK78* (C), *CsLRR-RLK295* (D), *CsLRR-RLK299* (E) and *CsLRR-RLK302* (F) in different tea accessions before (Con) and after herbivory (+ SE, *n* = 5). Colored dots on each plot represent individual data points from replicates. **(G-L)** Correlations between the expression of *CsLRR-RLK35* (G), *CsLRR-RLK55* (H), *CsLRR-RLK78* (I), *CsLRR-RLK295* (J), *CsLRR-RLK299* (K) and *CsLRR-RLK302* (L) genes, and larval weight. The Pearson’s product–moment correlation coefficient (*r*) and corresponding *P* values are provided. LJ43, Longjing 43.


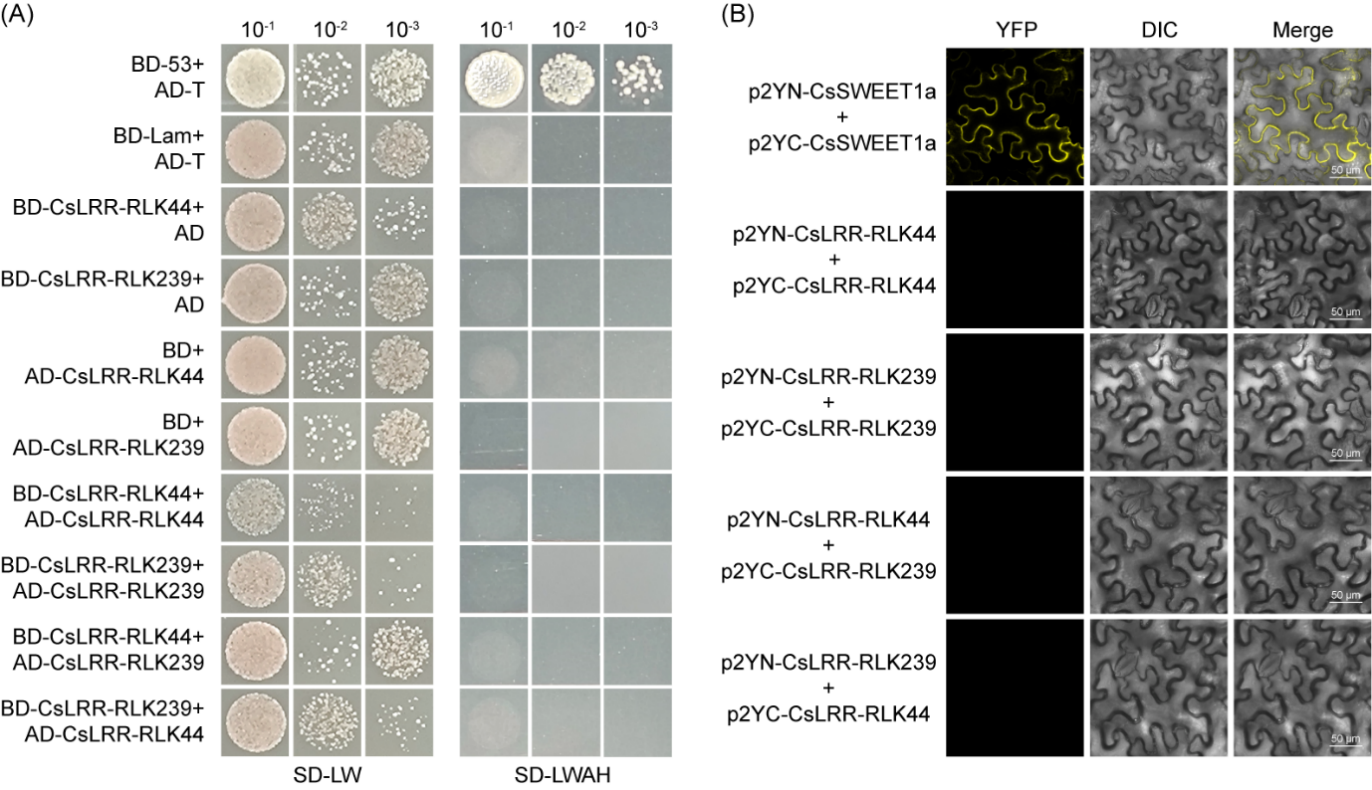


**Figure S3. CsLRR-RLK44 and CsLRR-RLK239 does not form homodimers with themselves or heterodimers with each other.** (A) Yeast two-hybrid (Y2H) assays were conducted to detect the interactions of CsLRR-RLK44 and CsLRR-RLK239, either with themselves or with each other. Transformed yeast cells were grown on SD/-Leu/-Trp (SD-LW) or SD/-Ade/-His/-Leu/-Trp (SD-LWAH) medium. The co-transformed of pGBKT7-53 (BD-53) and pGADT7-T (AD-T) served as the positive control, while pGBKT7-Lam (BD-Lam) and pGADT7-T (AD-T) served as the negative control. (B) Bimolecular fluorescence complementation (BiFC) assays were conducted to detect the interactions of CsLRR-RLK44 and CsLRR-RLK239, either with themselves or with each other. The vector *p2YN-CsSWEET1a* and *p2YC-CsSWEET1a* were used as a positive control pair for membrane-localized protein-protein interaction. Images displayed in sequence: YFP signal (yellow), differential interference contrast (DIC), and a composite overlay of both signals.


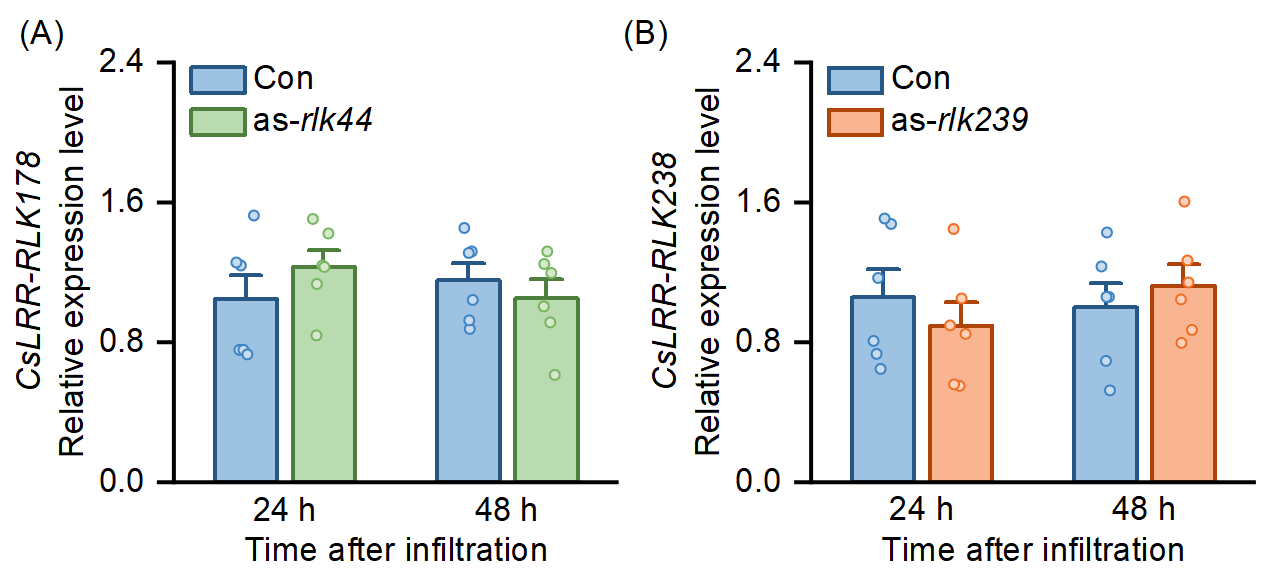


**Figure S4. Silencing of *CsLRR-RLK44* or *CsLRR-RLK239* does not affect the expression of their homologous genes.** Expression levels of *CsLRR-RLK178* and *CsLRR-RLK238* in *CsLRR-RLK44* (*as-rlk44*, **A**) and *CsLRR-RLK239*-silenced (*as-rlk239*, **B**) tea plants are shown (+ SE, n = 6). Colored dots on each plot represent individual data points from replicates.


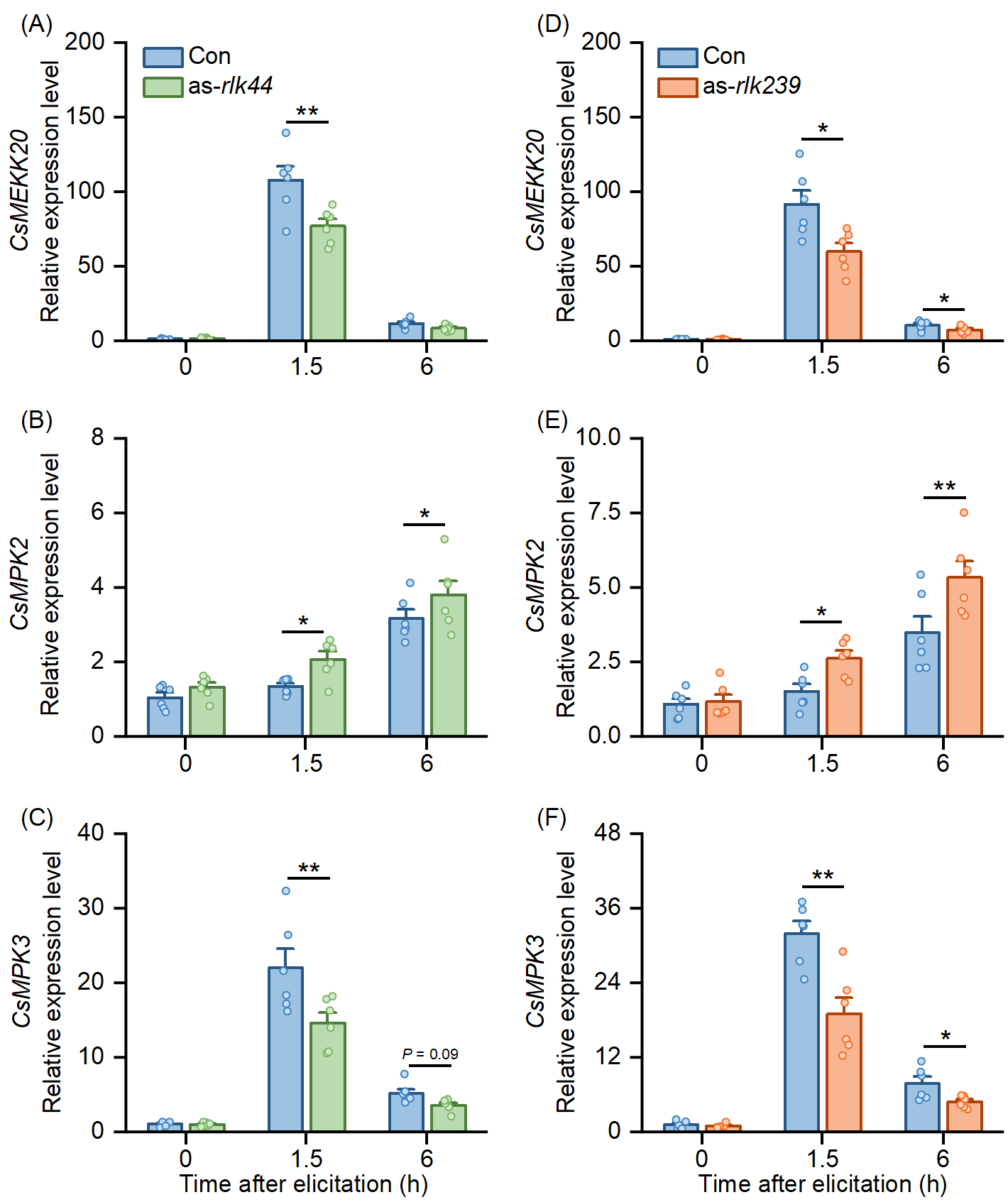


**Figure S5.** ***CsLRR-RLK44* and *CsLRR-RLK239* modulate the expression of defense-related MPKs. (A-C)** Expression levels of *CsMEKK20* (A), *CsMPK2* (B) and *CsMPK3* (C) in *CsLRR-RLK44*-silenced (as-*rlk44*) tea plants after herbivory (+ SE, *n* = 6). **(D-F)** Expression levels of *CsMEKK20* (D), *CsMPK2* (E) and *CsMPK3* (F) in *CsLRR-RLK239*-silenced (as-*rlk239*) tea plants after herbivory elicitation (+ SE, *n* = 6). Con, control. Colored dots on each plot represent individual data points from replicates. Asterisks indicate significant differences between treatments at different time points (two-way ANOVA followed by pairwise comparisons through FDR-corrected LSMeans; **P* < 0.05; ***P* < 0.01).


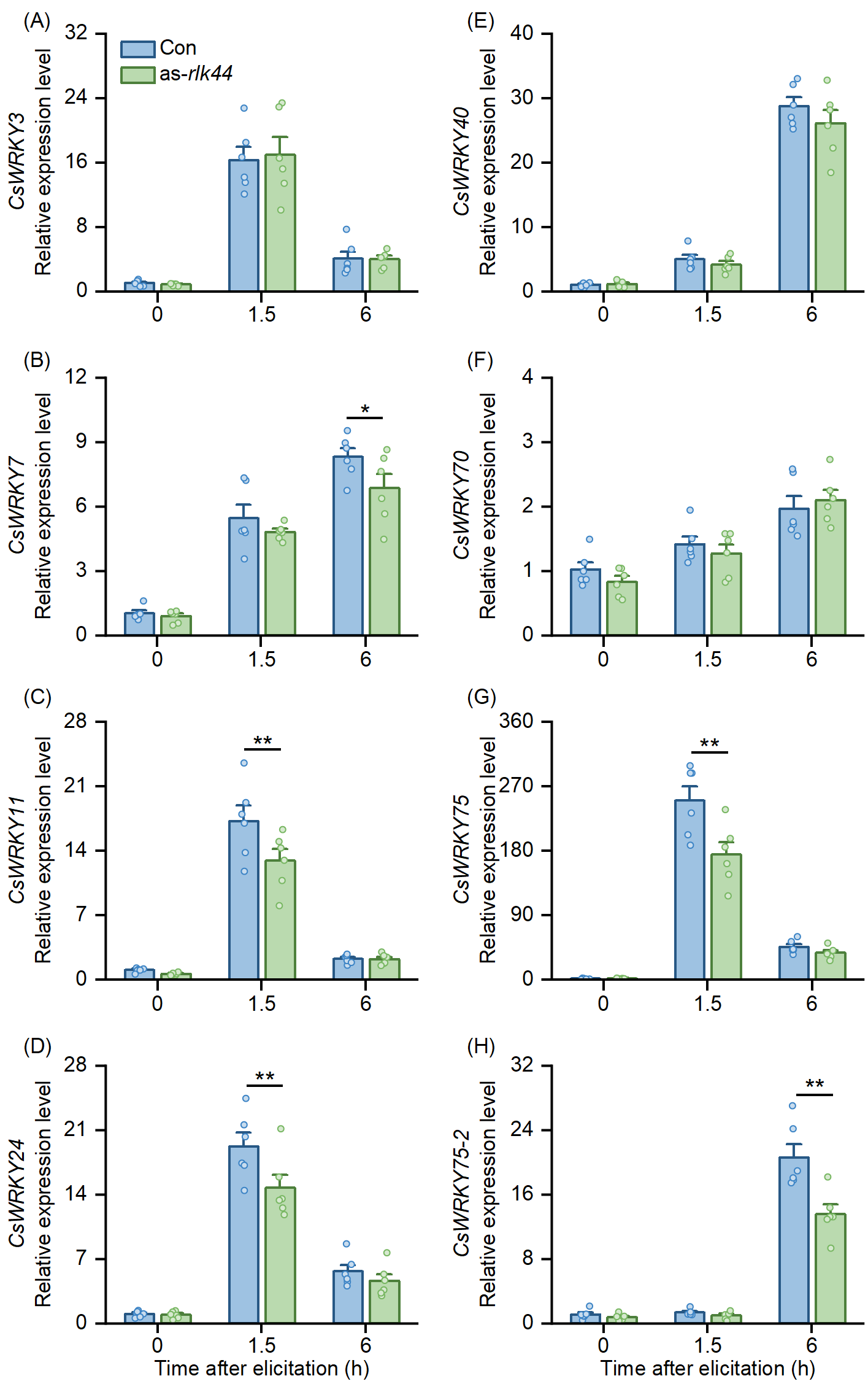


**Figure S6. *CsLRR-RLK44* regulates the expression of defense-related WRKYs.** Expression levels of *CsWRKY3* **(A)**, *CsWRKY7* **(B)**, *CsWRKY11* **(C)**, *CsWRKY24* **(D)**, *CsWRKY40* **(E)**, *CsWRKY70* **(F)**, *CsWRKY75* **(G)**, and *CsWRKY75-2* **(H)** in *CsLRR-RLK44*-silenced (as-*rlk44*) tea plants after herbivory (+ SE, *n* = 6). Con, control. Colored dots on each plot represent individual data points from replicates. Asterisks indicate significant differences between treatments at different time points (two-way ANOVA followed by pairwise comparisons through FDR-corrected LSMeans; **P* < 0.05; ***P* < 0.01).


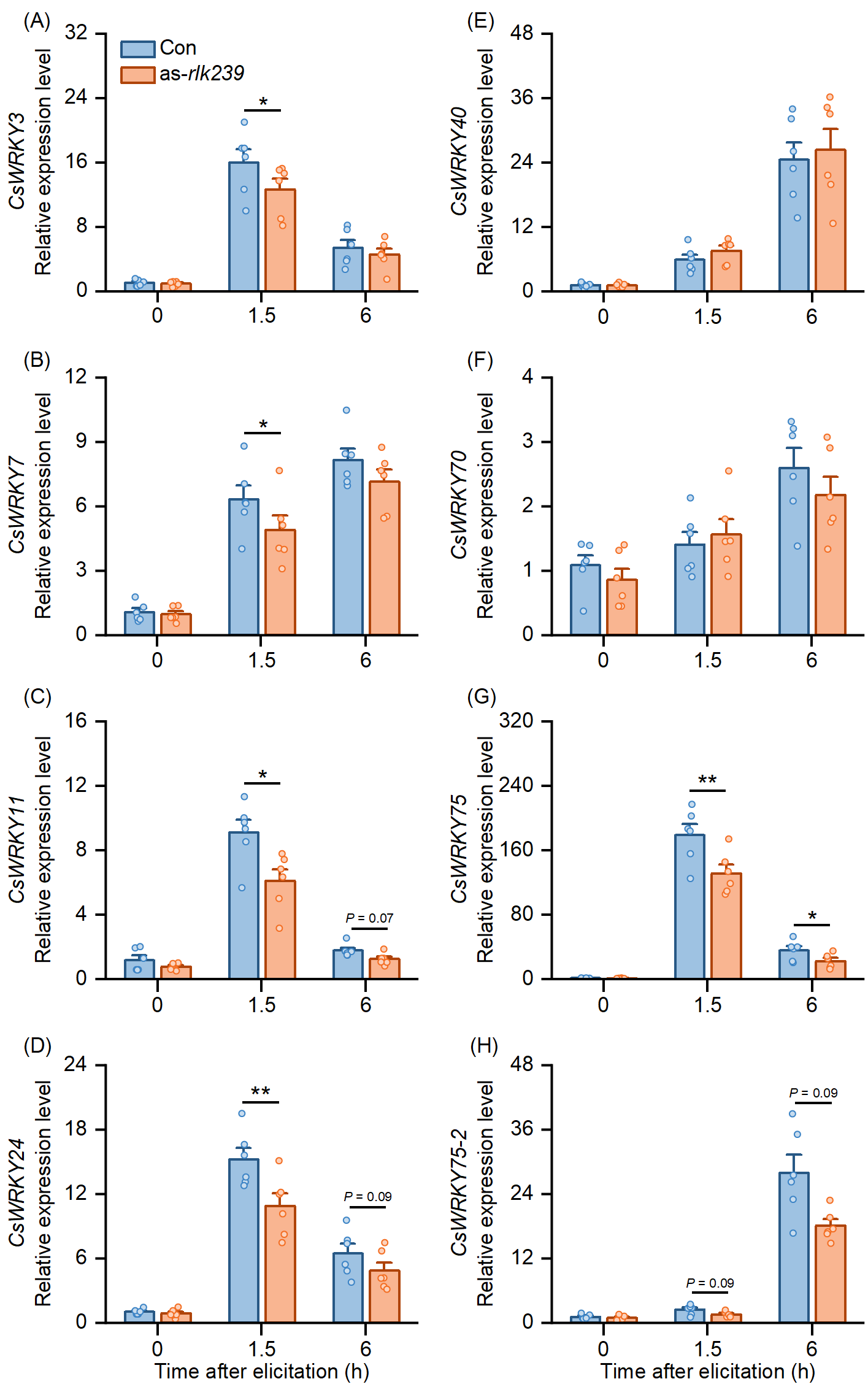


**Figure S7. *CsLRR-RLK239* regulates the expression of defense-related WRKYs.** Expression levels of *CsWRKY3* **(A)**, *CsWRKY7* **(B)**, *CsWRKY11* **(C)**, *CsWRKY24* **(D)**, *CsWRKY40* **(E)**, *CsWRKY70* **(F)**, *CsWRKY75* **(G)**, and *CsWRKY75-2* **(H)** in *CsLRR-RLK239*-silenced (as-*rlk239*) tea plants after herbivory (+ SE, *n* = 6). Con, control. Colored dots on each plot represent individual data points from replicates. Asterisks indicate significant differences between treatments at different time points (two-way ANOVA followed by pairwise comparisons through FDR-corrected LSMeans; **P* < 0.05; ***P* < 0.01).


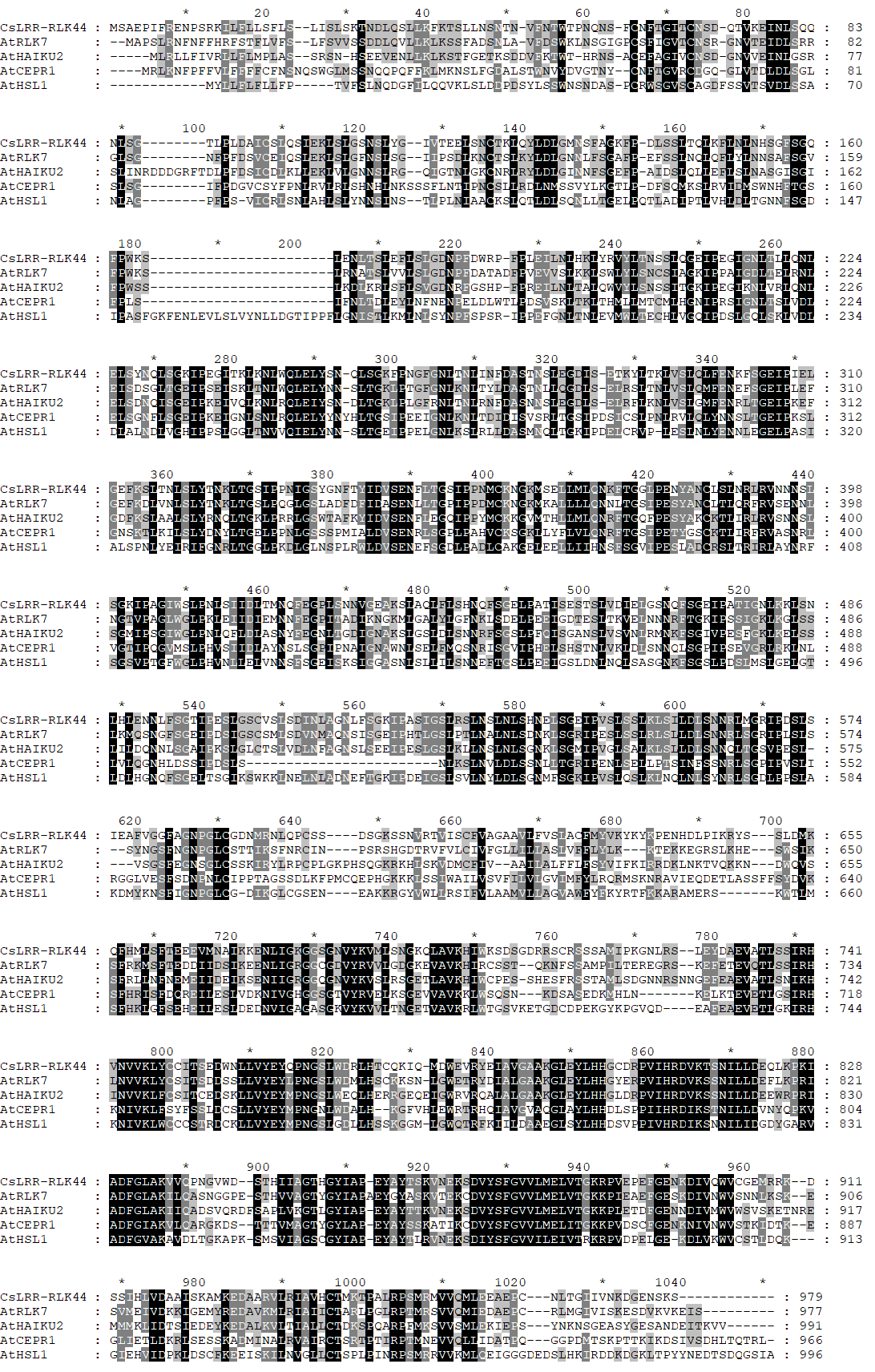


**Figure S8. Protein alignment of CsLRR-RLK44 with homologous proteins in Arabidopsis.** The amino acid sequence of CsLRR-RLK44 was aligned by ClustalW with homologous sequences of LRR-RLKs in Arabidopsis: AtRLK7 (AT1G09970.2), AtHAIKU2 (AT3G19700.1), AtCEPR1 (AT5G49660.1) and AtHSL1 (AT1G28440.1).


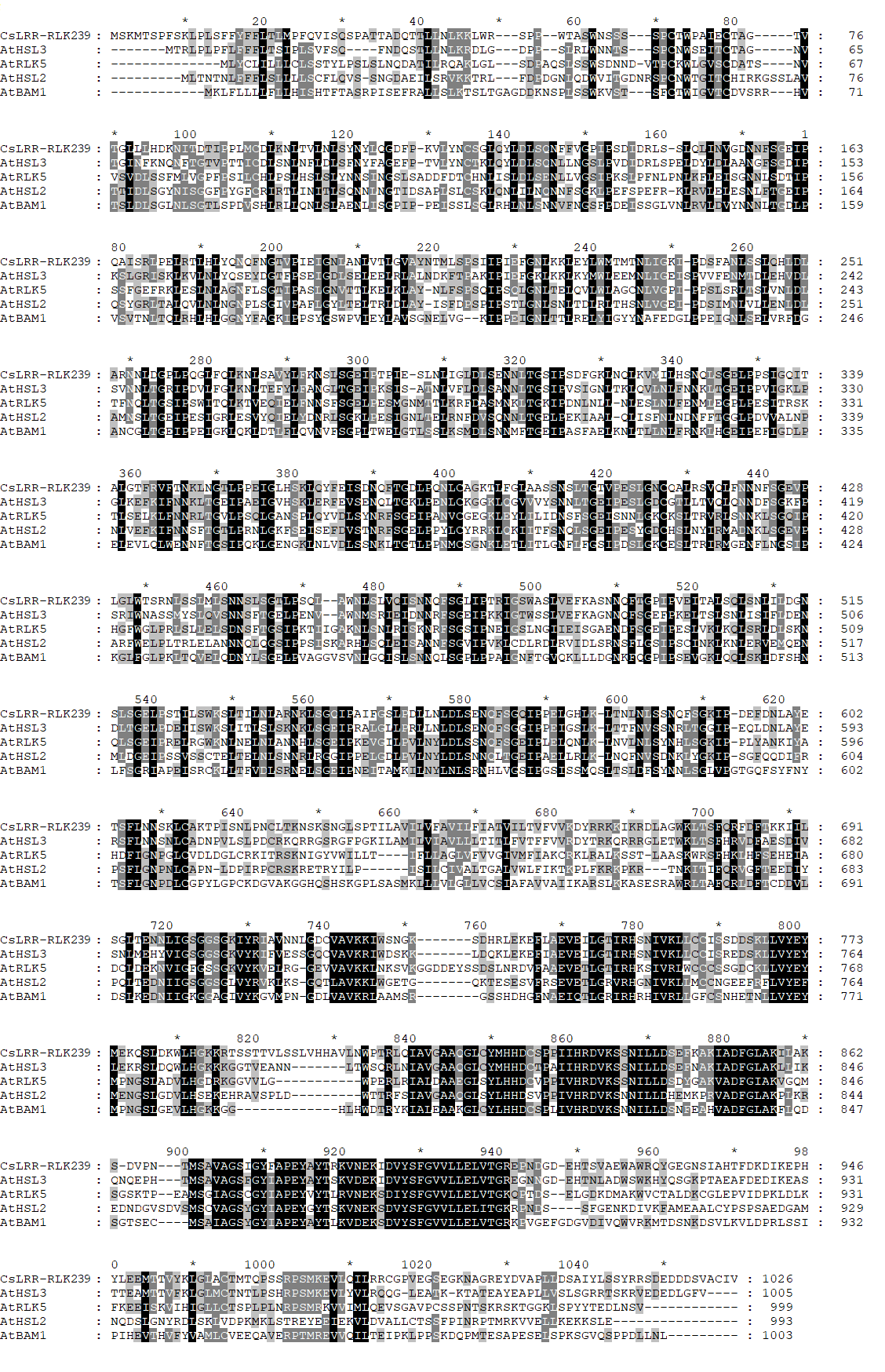


**Figure S9. Protein alignment of CsLRR-RLK239 with homologous proteins in Arabidopsis.** The amino acid sequence of CsLRR-RLK239 was aligned by ClustalW with homologous sequences of LRR-RLKs in Arabidopsis: AtHSL3 (AT5G25930.1), AtRLK5 (AT4G28490.1), AtHSL2 (AT5G65710.1), and AtBAM (AT5G65700.2).
